# Preliminary Data on Behavioral Profiles of Youth with Neurodevelopmental Disorders and Trauma

**DOI:** 10.3390/bs16020239

**Published:** 2026-02-08

**Authors:** Mathew C. Luehring, Leonora Ryland, Catherine Sanchez, Patrick W. Romani

**Affiliations:** 1 Pediatric Mental Health Institute, Children’s Hospital Colorado, Department of Psychiatry, University of Colorado Anschutz Medical Campus, 13123 East 16th Avenue, Aurora, CO 80045, USA; leonora.ryland@cuanschutz.edu; 2 All Minds Counseling, 1660 S Albion St. Suite 918, Denver, CO 80222, USA; catherine@allmindstherapy.com; 3 Division of Developmental Pediatrics, Children’s Hospital Colorado, Department of Pediatrics, University of Colorado Anschutz Medical Campus, 13123 East 16th Avenue, Aurora, CO 80045, USA; patrick.w.romani@ucdenver.edu

**Keywords:** neurodevelopmental disorders (NDDs), trauma exposure, challenging behaviors, self-injury, aggression, functional analysis, behavior analysis, Pediatric ACEs

## Abstract

Youth with neurodevelopmental disorders (NDDs) face an increased risk of trauma compared to their peers without NDDs, often leading to challenging behaviors such as self-injury, aggression, and property destruction. However, limited research exists on the behavioral profiles and treatment outcomes of youth with both NDDs and trauma. This study examines a sample of 21 youth with NDDs and trauma admitted to a specialized psychiatric unit in the Rocky Mountain region of the United States. A retrospective review of health records and admission data identified the most common target behaviors: negative vocalizations (95%), property destruction (62%), elopement (52%), and aggression (43%). Functional analyses indicated that escape was the most prevalent behavior function identified (43%), while 29% of the analyses yielded undifferentiated outcomes. Behavior analytic treatment packages incorporating differential reinforcement resulted in an average of 72% reduction from the baseline target behaviors. The average Pediatric ACEs score was 5 out of 10. The findings highlight the key behavioral patterns in this population and underscore the need for further research on effective interventions.

## 1. Introduction

Trauma is defined as “any disturbing experience that results in significant fear, helplessness, dissociation, confusion, or other disruptive feelings intense enough to have a long-lasting negative effect on a person’s attitudes, behavior, and other aspects of functioning” (American Psychiatric Association, 2022). These events can encompass a wide range of situations caused by human behavior (e.g., war, sexual assault, etc.) and/or nature (e.g., tornadoes, hurricanes, fires, etc.), as well as other situations. Within the literature, the term “adverse childhood experiences” (ACEs) has been used to describe a set of potentially traumatic experiences that occur during the developmental period prior to adulthood that have been found to consistently have long-lasting negative effects on various aspects of an individual’s physical, social, and psychological development. Traumatic events can occur at any point during someone’s life, though a significant amount of research has specifically documented the negative impacts of early childhood trauma experiences on behavior, relationships, cognition, and physical health (Dye, 2018). De Bellis (2001) proposed a developmental traumatology framework that suggests that chronic and severe trauma shifts from brain development and growth (a requirement during neurodevelopmental periods) to the survival mode, activating stress systems and leading to neurobiological changes. For example, it is well-regarded that complex, early trauma exposures cause long-term changes to neurobiology and brain functioning (Nemeroff, 2004). These changes may include memory and information processing (Briere & Scott, 2006) and modulating behavioral and emotional responses to stressors (Nemeroff, 2004). Regarding physical health, significant early childhood trauma and stress have been found to be a risk factor for various chronic diseases such as hypertension, obesity, sleep, and diabetes (Bader et al., 2007; Dong et al., 2004; Greenfield & Marks, 2010). There have been conflicting studies on whether the number or type of ACEs is most influential to development and trauma (Nelson & Gabard-Durnam, 2020). For example, McLaughlin, Sheridan et al. (McLaughlin & Sheridan, 2016; McLaughlin et al., 2014; Sheridan & McLaughlin, 2014) reported that physical abuse affects the brain differently from neglect. Other studies have shown a cumulative effect of ACEs on the increased risk of various psychopathologies in adulthood. For example, identification of four or more ACEs has been found to increase the odds ratio for developing panic disorders, depression, anxiety, and hallucinations (Anda et al., 2006).

### Trauma and Neurodevelopmental Disorders

Neurodevelopmental disorders (NDDs) are a group of conditions that usually begin during the developmental period and involve delays or impairments in personal, social, academic, or occupational functioning. NDDs encompass many disorders, including autism spectrum disorders (ASD), intellectual developmental disorder (IDD), attention deficit hyperactivity disorder (ADHD), communication disorders, and learning disorders, among others. Prevalence estimates of NDDs in the general population suggest that 5–20% of children will be diagnosed with an NDD (Patel & Merrick, 2020). Worldwide diagnostic prevalence of NDDs has been on the rise in recent decades due to a combination of factors, including improved early detection/screening, more accurate diagnostic procedures, and increased awareness (Olusanya et al., 2018; Zablotsky et al., 2019). In conceptual models of trauma (and more specifically, diagnoses of post-traumatic stress disorder, PTSD), there are a variety of factors that influence the development of trauma diagnoses. Christoffersen and Thorup (2024) described this in their conceptual model, which featured familial (e.g., traumatic stress), environmental (e.g., community violence), and individual factors (e.g., diagnoses, temperament, etc.), which all represent factors that influence how an individual who experiences trauma may differentially affect how this trauma presents and is diagnosed. NDDs represent individual factors that can lead to more frequent stress or conflict and can also influence familial stress, which may then predispose children with NDDs to higher rates of trauma diagnoses.

Research has identified a bidirectional relationship between ACEs and NDDs, suggesting that ACEs may increase the risk of developing certain NDDs and that neurodiverse individuals^1^ are more likely to experience ACEs (Jangid et al., 2025). For example, research has shown that individuals with ASD have twice the likelihood of encountering an ACE compared to individuals without ASD (Hartley et al., 2024). Similarly, Vervoort-Schel et al. (2021) found that 81.7% of children with ID experienced at least one ACE, compared to 63.9% of the general population (Swedo, 2023). According to Takeda et al. (2024), individuals with ASD were four times more likely to experience at least four ACEs compared to individuals without ASD. Brain imaging studies have found that early adversity was linked to changes in the amygdala volume among children with ASD, suggesting heightened neurobiological sensitivity to stress (Kuenzel et al., 2025), with the same changes not observed in children who were developing typically. Additionally, Kuenzel et al. found that changes in the amygdala volume were more significant for autistic children compared to adolescents, highlighting that early adversity does differentially affect individuals based on their age. Another study found that people with ASD who went through severe childhood adversity had changes in the right superior temporal gyrus (the brain region linked to sensory sensitivity). These changes suggest that these individuals with NDDs may be more affected by early trauma, and their heightened reactions to such things as sounds, lights, or touch could make those effects even stronger. Other research has suggested that differences in how autistic individuals perceive and process information, such as traumatic events, can further affect how these events impact individuals with ASD. Finally, individuals with NDDs may be at higher risk for bullying or child maltreatment (e.g., abuse, neglect).

Diagnosing trauma in children and adolescents with NDDs can be challenging and requires multi-faceted approaches. One challenge with diagnosing trauma in this population lies in the concept of diagnostic overshadowing. Diagnostic overshadowing is the notion that a bias from a clinician leads to a failure to detect a disability or symptoms because its features are attributed to another, primary, disability or symptom (American Psychiatric Association, 2022). For neurodiverse individuals, trauma symptoms may be overlooked as symptoms of their neurodevelopmental disorder and, as a result, may negatively impact their ability to receive a diagnosis of trauma (e.g., PTSD), leading to potential delays in treatment. For example, for an individual with a diagnosis of ASD who has been experiencing more significant symptoms of irritability, concentration difficulties, relational challenges, social isolation, or repetitive or stereotypical behaviors, without a more thorough review of changes to these symptoms and further assessment into the area of trauma, these behaviors may be assumed to be due to the ASD diagnosis. Another challenge with diagnosing trauma in NDDs lies in the social communication difficulties that individuals with NDDs can possess and that for some individuals, self-report may either be unreliable or could be limited (Hoover, 2020). This can be even more difficult for young children or those with significant communication impairments. Finally, there is a limited number of trauma screening and assessment tools that are validated for use with individuals with ASD and/or ID, creating issues with evidence-based assessments for trauma in this population. All together, these difficulties present challenges for the assessment of comorbid trauma for individuals with NDDs.

Without accurate diagnoses for trauma for neurodiverse individuals, delays to trauma-specific treatments may occur. Further, trauma-informed approaches may not be incorporated without a detailed understanding of potential traumatic experiences or events for individuals. Little is known about the extent to which trauma impacts progress with evidence-based treatments for youth with NDDs. According to the substance use disorder (SUD) literature, individuals with substance use may experience a higher risk of relapse (Norman et al., 2007) and may have poorer outcomes with less improvement (Ross et al., 2021). According to the trauma literature on military adults, individuals with significant trauma experience poorer treatment adherence, or higher rates of attrition, which then negatively affect their treatment progress and outcomes (Berke et al., 2019). The impact of trauma on treatment outcomes has not received much attention within the area of NDDs, but given the literature on other disorders and populations, it is plausible to consider whether the presence of trauma could negatively impact treatment outcomes and long-term progress.

While some previous literature focused on profiles of youth with NDDs and trauma (see Cook & Hole, 2021 for a scoping review), little current literature exists on more specific behavioral profiles for these children and youth, especially those with severe and/or complex behavioral problems. Identifying behavioral profiles for these individuals is beneficial for a variety of reasons. First, identifying patterns in behavioral profiles in research can improve clinical practice by enhancing diagnostic accuracy and guiding treatment decisions (Mosavi & Santos, 2024). These improvements may also lead to advancements in research and improve collective understanding of treatment outcomes, including identifying latent profiles, such as individuals who may respond differently to different interventions. All these factors may help contribute to improved precision mental health care (Moggia et al., 2024). Precision mental health care emphasizes the use of “personalized data-driven strategies to optimize outcomes for a particular patient” (Moggia et al., 2024, p. 611). In order to establish precision mental health care, large datasets from patient populations are needed to guide assessments and generate recommendations and interventions. From a research perspective, these large datasets are necessary to identify prognostic factors and moderators that affect treatment outcomes for specific populations, which will then allow clinicians to identify effective individualized interventions that lead to better outcomes for individuals. Several large-scale review studies exist that explore results and structure of structured behavioral assessments, participant demographics (e.g., disorders, disabilities, etc.), and settings (see reviews by Hanley et al., 2003; Beavers et al., 2013; and Melanson & Fahmie, 2023). These reviews provide detailed information that has led the field of applied behavior analysis (ABA) in reviewing the gold-standard behavioral assessment methodology known as functional analysis (see Iwata et al., 1994). In these reviews, diagnoses of trauma were not included for analysis, limiting the generalizability of these reviews for individuals with severe and/or challenging behaviors, NDDs, and trauma.

As highlighted above, there is a significant gap in the existing literature exploring both the characterization of neurodiverse youth with trauma and the effects that trauma has on these youth. To address this, the current study seeks to explore several aspects related to youth with NDDs and trauma. First, we sought to explore behavioral profiles of youth with NDDs and trauma who were admitted to a specialized psychiatric unit to better characterize this sample and extend previous literature to support generalizability in this population. Secondly, we looked at the outcomes of behavioral therapy for these participants to understand the impact of trauma to better support considerations when working with youth with NDDs and trauma. Given the exploratory nature of this study, we hope to shed light on behavioral profiles that can help characterize youth with NDDs and comorbid challenging behaviors and do not offer specific hypotheses regarding this aim. We hypothesized that trauma would negatively impact behavioral therapy progress for the participants in the current study.

## 2. Methods

### 2.1. Participants

This study examines a sample of 21 youth with NDDs and trauma admitted to a specialized psychiatric unit in the Rocky Mountain region of the United States due to severe and/or challenging behavior. An archival review of the participants admitted to a psychiatric special care unit between April 2021 and December 2024 was conducted. Inclusion criteria: participants aged 4–17 years old with an identified primary or secondary diagnosis of trauma. Initially, 26 participants met the inclusion criteria. If the participants did not have direct behavioral data collected through individual treatment sessions with an experimental demonstration of the effect of the independent variable utilizing a single-case design, they were excluded. Ultimately, 21 participants were included in this research project; full demographics of those participants are available in Table 1. Data extraction included a thorough review of medical records, behavioral assessment and treatment details, and behavioral reduction data calculated for all the participants admitted. The current study was reviewed and determined to be exempt from Institutional Review Board (IRB) oversight in accordance with institutional policies and federal regulations.

#### Diagnosis

Upon admission to the psychiatric unit, the primary and secondary diagnoses of each participant were identified through a psychiatric evaluation by an interdisciplinary team comprised of a psychiatrist, a psychologist, and a social worker. These psychiatric evaluations included a review of previous symptoms, presenting concerns, and familial history, among other focuses. At times, these diagnoses included structured screenings and evaluation tools (e.g., Screen for Child Anxiety Related Emotional Disorders (SCARED; Birmaher et al., 1997), Revised Child Anxiety and Depression Scale (RCADS; Chorpita et al., 2000)), while other diagnoses were made using clinical judgment and informal assessments. All the participants were admitted with previous NDD diagnoses. Diagnostic reports were requested upon admission and were often verified through previous testing with a licensed psychologist that included either a medical diagnosis or a school psychologist that included an educational disability/classification.

### 2.2. Procedures

#### 2.2.1. Review of Medical Records

Utilizing an electronic medical record system, the researchers reviewed details from those participants who met the inclusion criteria. This review included the inpatient or partial hospitalization program (PHP) chart notes, daily progress notes (including participant responses to treatment (represented by the combined rate of target behaviors during treatment sessions) and progress across individual and group treatments).

#### 2.2.2. Behavioral Assessment

During admission to the psychiatric program (either inpatient or partial hospitalization), each participant assented to behavioral assessments, including indirect measures (surveys, questionnaires, etc.) and direct assessments (functional analysis (FA), structured familial observations, concurrent operant assessment, etc.). Indirect (questionnaires and surveys with the participants and families) assessments were conducted initially to support generating and testing relevant FA conditions. These included conducting interviews with caregivers about the various scenarios that the participants have struggled with at home or in the community setting (for example, attention, tangible, escape, and being alone). Direct (i.e., FA) assessments were conducted to confirm the hypothesized functions of behavior. FAs followed the conditions described by Iwata et al. (1994). The sessions were 5 min in duration and included the following conditions: free play/control, attention, escape, and tangible. During the free play condition, a therapist was present and provided the participant with non-contingent access to preferred items and attention every 30 s in the form of praise. This condition sought to serve as a control condition, to which comparisons with the other test conditions could be made. During the attention condition, the participants had access to moderately preferred items and 2 min of access to undivided attention prior to the session beginning. At the start of the session, the therapist notified the participant that they were unavailable and made themselves busy. The therapist provided brief attention/reprimands contingent on target behaviors. This condition sought to test for target behaviors maintained by positive social reinforcement (i.e., access to attention). During the escape condition, the therapist delivered demands identified by the participant’s caregiver during interviews that may be likely to evoke disruptive behaviors. The therapist provided a brief (30 s) break from demands contingent on target behaviors. This condition sought to test for target behaviors maintained by negative reinforcement (i.e., escape from demands). During the tangible condition, the therapist provided the patient with a highly preferred item for 2 min. At the beginning of the session, the therapist removed access to the preferred item and provided brief (30 s) access to the item contingent on target behaviors. This condition sought to test for target behaviors maintained by positive social reinforcement (i.e., access to the preferred items/activities). FAs were continued until differentiation occurred between the conditions. Graphs were visually analyzed using structured criteria described by Roane et al. (2013) to confirm the hypothesized function(s) maintaining target behaviors.

#### 2.2.3. Behavioral Treatment Details

Most participants engaged in the treatment focused on decreasing target behaviors and increasing adaptive behaviors, such as those related to appropriate communication with functional communication training (FCT) aligned with functional replacement behaviors. For instance, when results of the FA determined that the participant’s target behaviors were maintained by escape from demands, then FCT for escape intervention that prioritized the practice of requesting breaks over non-preferred demands and increasing tolerance for waiting for breaks (Heath et al., 2015). Several participants engaged in therapeutic work during individual treatment sessions to support generalization of emotional regulation strategies during group treatment sessions (Carruthers et al., 2020). This occurred within the context of an FCT escape routine. Most participants’ (95.2%, *N* = 20) behavioral interventions followed a reversal design, notably ABAB. This involved implementation of treatment, then a return to the baseline (assessment sessions), then a return to treatment.

#### 2.2.4. Behavioral Reduction Data

Calculation of the reduction of target behaviors was performed based on the baseline (an average of the rate of target behaviors observed during FA) and the average rate of target behaviors during the last five sessions of treatment during admission. If there were zero rates of target behaviors during the baseline, then there is “n/a” for behavioral reduction. If there was an increase in the target behaviors, then a “0%” reduction in the target behaviors was identified (Wacker et al., 2011).

#### 2.2.5. PEARLS

The Pediatric ACEs and Related Life Events Screener (PEARLS) is a comprehensive screening tool used to measure the exposure to adverse childhood experiences (ACEs) and other traumatic stressors in children and adolescents aged 0 to 19 (Thakur et al., 2020). In each version of the assessment, qualitative data are collected regarding the child’s or adolescent’s exposure to ACEs, as well as additional risk factors that may impact physical and psychological well-being throughout an individual’s lifetime (Ye et al., 2023). This study utilized both the PEARLS child and teen assessments, assigning the appropriate assessment to each study participant as indicated by the participant’s age. The PEARLS child assessment is a 17-item assessment used for children aged 0 to 11. The PEARLS teen assessment is a 19-item assessment utilized for adolescents aged 12 to 19. Each assessment was completed by the third author via a thorough review of the identified participant’s medical records regarding the participant’s life experiences. Retrospective chart review of ACEs was used in previous studies (e.g., Yamagishi et al., 2022), as well as with adults to review the ACEs encountered in childhood to predict health outcomes in adults (Baldwin et al., 2021; Reuben et al., 2016). PEARLS Part 1 focuses on such ACEs as abuse, neglect, and/or household dysfunction. PEARLS Part 2 looks more closely at such risk factors as social determinants of health (Thakur et al., 2020).

#### 2.2.6. Associations Between ACEs and Problem Behavior Reduction

To determine whether associations between ACEs and reductions in problem behaviors existed, a point-biserial correlation was calculated. ACEs were a categorical variable with two levels (i.e., groups). ACEs were dichotomized to two groups (four or more ACEs and less than four ACEs). These groups were determined as four or more ACEs fall within the “high risk” category (Office of the California Surgeon General, 2020). Problem behavior reduction was a quantitative variable depicted as a percentage of reduction in behavior between the treatment and the baseline.

#### 2.2.7. Reliability

The second researcher conducted interrater reliability for 33% (*N* = 7) of the patients’ charts for PEARLS scores. The total scores for both Part 1 and Part 2 of the PEARLS were calculated and compared. The interrater reliability was calculated by each rater summing the total number of “Yes” responses in Part 1 and the total number of “Yes” responses in Part 2 for all the patients and dividing the larger number by the smaller number and multiplying by 100 for each Part 1 and Part 2. The interrater reliability for Part 1 of the PEARLS was 97.1% and 100% for Part 2.

Multiple researchers conducted visual analysis of FA graphs, utilizing structured criteria established by Roane et al. (2013) to determine hypothesized functions of target behaviors. Based on the criteria, FA results were determined to be “undifferentiated” if there were not clearly elevated session types (e.g., attention, tangible, escape) or without an upward trend. Across two researchers, interrater reliability was collected for 100% of all the FA graph interpretations, and the reliability was 96.0%.

### 2.3. Power Analysis

To determine the appropriate sample size for identifying a significant point-biserial correlation (*r_pb_*) between four or more ACEs and reduction in problem behavior, a power analysis using G*Power 3.1 (Faul et al., 2007) was conducted with parameters set to an expected effect size of *r_pb_* = 0.30, a desired power of 0.80, and an alpha level of 0.05. Across the 21 participants, there were 26 datasets included in total, with 26 datasets required to detect a moderate effect size.

### 2.4. Data Analysis

Statistical analyses were calculated using IBM SPSS Statistics version 31.

## 3. Results

### 3.1. Demographic Information

As depicted in Table 1, demographics of the participants included 38.1% (*N* = 8) individuals assigned female at birth, 61.9% (*N* = 13) assigned male at birth, and 9.5% (*N* = 2) transgender. The participants’ racial identities were as follows: 4.8% (*N* = 1) African American, 85.7% (*N* = 18) Caucasian, 4.8% (*N* = 1) Asian American Pacific Islander, and 4.8% (*N* = 1) multiracial. In addition, 4.8% (*N* = 1) of the participants identified as Hispanic, and 95.2% (*N* = 20) identified as non-Hispanic. The participants’ gender was as follows: 38.1% (*N* = 8) assigned female at birth, 61.9% (*N* = 13) assigned male at birth, and 9.5% (*N* = 2) transgender. A review of admission diagnoses revealed that 42.9% (*N* = 9) of the participants had a primary diagnosis of trauma; 57.1% (*N* = 12) had a secondary diagnosis of trauma. Full details of the participant demographics are available in Table 1.

### 3.2. Target Behaviors Identified Through Behavioral Assessment

**Figure 1:**
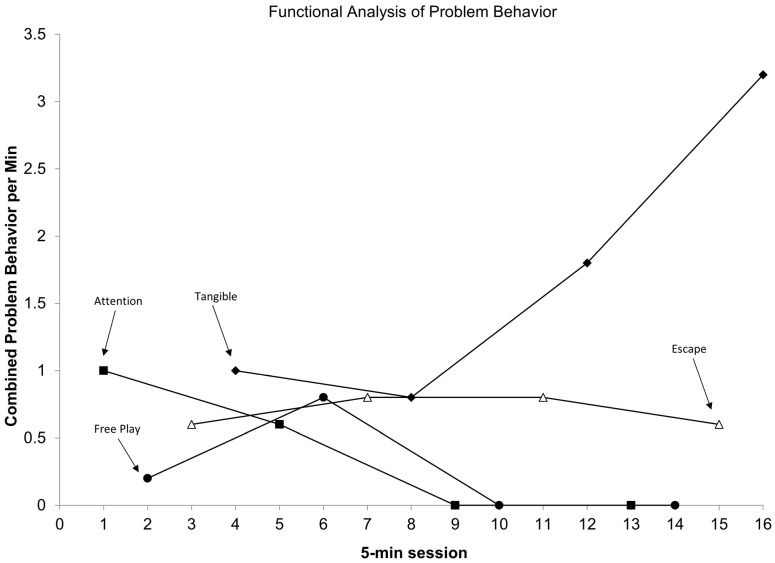
Sample of a participant’s functional analysis graph. Functional analysis graph for participant #6. Sessions are found on the *x*-axis, with the rate of target behaviors (responses per minute) found on the *y*-axis. Closed black circles depict free play conditions. Closed black squares depict attention conditions. Open white triangles depict escape conditions. Closed black diamonds depict tangible conditions.

Target behaviors were identified for each participant through chart review and indirect interviews with caregivers as part of the participant’s standard of care during their admission. A retrospective review of health records and admission data identified the most common target behaviors across all the participants included: negative vocalizations (95.2%, *N* = 20), property destruction (61.9%, *N* = 13), elopement (52.4%, *N* = 11), aggression (42.9%, *N* = 9), self-injurious behaviors (19.0%, *N* = 4), and somatic symptoms (4.8%, *N* = 1). As a note, the participants could have more than one target behavior identified for reduction during admission. Functional analyses indicated that escape was the most prevalent behavior function identified (42.9%, *N* = 9), followed by attention (23.8%, *N* = 5), and tangible (23.8%, *N* = 5). Across all the participants, 28.6% (*N* = 6) of the analyses yielded undifferentiated outcomes, and no FA results indicated automatically maintained target behaviors. These undifferentiated outcomes were often a result of zero or near-zero rates of target behaviors (*N* = 6). For the purposes of this research project, indirect behavioral assessments were not used to hypothesize the functions of behavior. Figure 1 depicts an example of a graph for a participant whose FA resulted in combined escape and tangible functions.

### 3.3. Design and Response to Individualized Intervention

**Figure 2:**
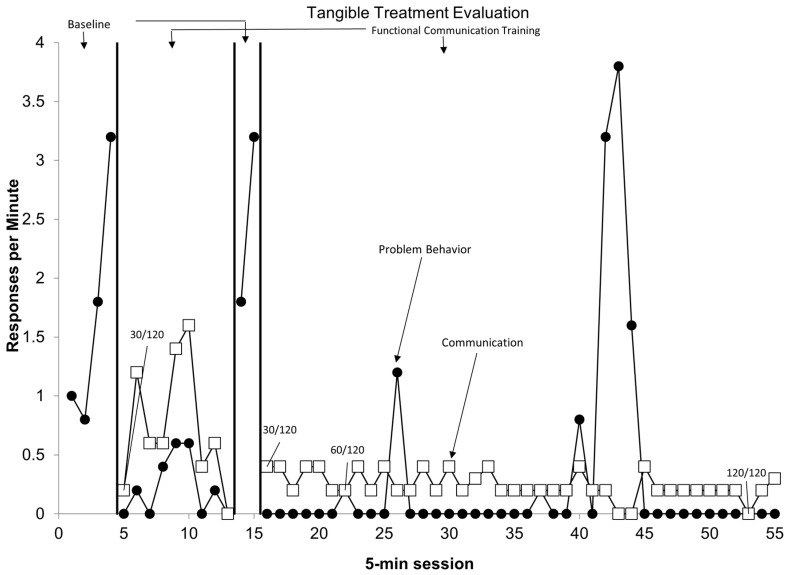
Sample of a participant’s treatment graph. Treatment graph for participant #6. Sessions are found on the *x*-axis, with the rate of target behaviors (responses per minute) found on the *y*-axis. Closed black circles depict combined problem behaviors. Open white squares depict functional communication responses.

Behavior analytic treatment packages incorporating differential reinforcement (e.g., FCT) resulted in an average 72.4% reduction in the baseline target behaviors. As can be seen in Table 2, across the various treatment packages, there was an average of 66.8% reduction in FCT escape, 100.0% reduction in FCT tangible, and 65.4% reduction in FCT attention. Figure 2 depicts an example of an FCT tangible routine for a patient who demonstrated a 100% reduction in target behaviors.

### 3.4. PEARLS

Table 2 depicts the PEARLS scores across both Part 1 (ACEs) and Part 2 (SDOH). The average Pediatric ACEs scores were 5.0 for Part 1 and 2.2 for Part 2.

### 3.5. ACEs and Reduction in Problem Behavior

Results of the point-biserial correlations revealed that the relationship between four or more ACEs and the reduction in problem behavior was not significant, *r_pb_*(24) = 0.002, *p* = 0.99.

## 4. Discussion

The current study sought to extend the previous literature on understanding comorbid trauma in children and adolescents with NDDs by establishing behavioral profiles of youth with NDDs. While the previous literature identified high comorbidities, our research sought to characterize individuals with NDDs and comorbid trauma, examining behavioral profiles and common factors relevant during their behavioral assessments and treatments. A retrospective design was used to identify 21 participants admitted to a specialized psychiatric hospital unit for youth with NDDs over a period of 3.5 years. The results indicated that most patients displayed negative vocalizations (95.2%), property destruction (61.9%), or elopement (52.4%). Higher severity target behaviors, such as aggression and self-injury, occurred less often, in 42.3% and 19.0% of the sample, respectively. Previous literature has identified negative vocalizations (24.8%), property destruction (20.9%), and elopement (12.3%) as being some of the less frequent behavioral topographies, with aggression (55.2%) and self-injury (41.7%) being the most common (Melanson & Fahmie, 2023). Other similar research on youth within these specialized psychiatric units found results consistent with the present study, with property destruction (84.3%) and negative vocalizations (78.1%) being the more common topographies, while aggression (81.4%) was also reported to occur more frequently (Romani et al., 2021). These differences might be accounted for by the difference in population studies (e.g., general population versus those admitted to specialized psychiatric units). Escape was the most common function identified through FAs (42.9%), consistent with other studies (Romani et al., 2021; Melanson & Fahmie, 2023), followed by attention (23.8%) and tangible (23.8%). Of note, undifferentiated FAs were found in 28.6% of the sample within the current study. No FAs identified automatically maintained target behaviors (0%). Our results for the identified functions differed, with 28.6% of the FAs in the current study resulting in undifferentiated outcomes, compared to others (e.g., 18.8% in Romani et al., 2021; 8.9% in Melanson & Fahmie, 2023; 8.3% in Beavers et al., 2013; and 4.1% in Hanley et al., 2003). It is plausible to suggest that the comorbidity between NDDs and trauma complicates FA results, leading to higher rates of undifferentiated outcomes, though additional research is needed to confirm or refute this. Some research has suggested that repeated exposure to establishing operations may lead to more escalations during assessments (Jessel et al., 2023). Due to the limited admission lengths (e.g., 10–12 days) in the current study, additional more in-depth assessments were not sought out, potentially limiting the identification of functions through further modifications of the FAs (see Hagopian et al., 2013 and Roscoe et al., 2015 for examples). Another potential explanation is that multiple topographies of target behaviors were identified for inclusion in the FA and, potentially, different reinforcers were maintaining different target behaviors, ultimately leading to undifferentiated results (Derby et al., 1994). However, FAs for the participants in the current study were continued until differentiation was found, or the results were undifferentiated, and the supervising psychologist decided to stop assessments.

Within our sample, 42.9% of the participants had a primary diagnosis of a trauma- or stressor-related disorder (e.g., PTSD). Scores from the PEARLs indicated average ACEs (Part 1) scores of 5.0 for the sample, with the average social determinants of health scores of 2.2. Traditionally, PEARLS ACEs scores of 0 are considered “low risk,” scores of 1–3 are considered “intermediate risk,” and scores of 4 or more are considered “high risk.” Research has shown a cumulative effect of ACEs, with an increased risk of developing NDDs (Jangid et al., 2025). Additionally, children with four or more ACEs face a significantly higher risk of both mental and physical health problems (Baldwin et al., 2021). Our results highlighted the significant number of ACEs identified within the current sample, which fall within the “high risk” category. Additional studies are needed to continue to identify base rates of ACEs for neurodiverse children and adolescents. The current study did not find a relationship between the number of ACEs and reductions in problem behavior. This may be in part due to the small sample size (adequate power to identify moderate effect sizes only). It may also be plausible that the number of ACEs does not have an effect on reductions in problem behavior, though additional research is needed. However, this is a recommended area of future study and could have particularly beneficial clinical implications. The results of the current study provide preliminary evidence of behavioral profiles for a small sample of youth with NDDs and trauma. These data are important to characterize samples of youth with NDDs and comorbid trauma to better understand this population. Additional research is needed to explore how trauma may differentially affect behavioral treatment outcomes, as well as to continue to characterize this sample of youth with NDDs and trauma.

Despite the contributions of the current study, several limitations exist. First, the sample of participants was taken from individuals who were admitted to a specialized psychiatric unit. This unit included individuals who were admitted to both inpatient and partial hospitalization levels of care. These levels of care are required for individuals who are experiencing a behavioral health crisis, necessitating intensive interventions, and do not represent community-level participants. Because of this, external validity may be limited as the sample represents youth with more significant behavioral challenges. Additionally, the current study reviewed results for only 21 youth with NDDs and trauma. This small sample size limits the types of analyses that may be conducted and any subgroup analyses (e.g., gender, age, race, etc.). The current study was powered to detect a moderate effect size. Given this, it is possible that a small effect size was present and the current study was underpowered to identify this small effect size. Despite this, the current study sought to provide a preliminary description of the characteristics of youth with NDDs. Additional research with larger datasets is needed to continue to explore the association between trauma and behavioral profiles and behavioral outcomes for this population, as well as to conduct any secondary analyses for moderators, mediators, or extraneous variables. Thirdly, there are limitations with the use of the PEARLS ACEs scores obtained through a retrospective chart review. Ideally, a chart review would be supported with a clinical interview to confirm potential ACEs and identify any ACEs missed through chart review. However, poor reliability has been found between prospective and retrospective review of ACEs (Reuben et al., 2016; Naicker et al., 2017). Future studies could begin with a chart review and confirm potential ACEs through caregiver interviews/rating scales. Finally, comprehensive diagnostic evaluations were not conducted to verify previous diagnoses for these participants, as the sample was collected through retrospective chart review. Future studies could independently verify and ensure the reliability of diagnostic results. This also limits some of the conclusions made with respect to certain diagnostic groups.

## 5. Conclusions

Limitations notwithstanding, the current study provides valuable preliminary information regarding behavioral profiles of youth with NDDs and comorbid trauma. Characterizing this population and identifying behavioral profiles for these youth is an essential preliminary step in improving individualized, tailored interventions, which have important clinical implications. The current study does highlight some challenges related to diagnosing trauma within the population of children and adolescents with NDDs. Limited, valid screening tools exist for this population, and more research is certainly needed to validate tools for use. Screening, assessing, and understanding trauma is a crucial part of trauma-informed care for youth with NDDs. Rajaraman et al. (2022) outline several considerations for trauma-informed applied behavior analysis (ABA), including screening for trauma and incorporating trauma-informed approaches to ABA. Given the higher risk of trauma within this population, clinicians should screen for potential trauma and always use trauma-informed approaches to ensure ethical care for individuals.

## Figures and Tables

**Table 1:** Patient demographics.

ID#	Diagnoses	Age	Gender	Race and Ethnicity
1	DMDD, PTSD, ADHD, MiID	12	Male	White, nonH
2	PTSD, ADHD, ASD, DBD	10	Female	White, nonH
3	PTSD, ADHD, ASD	9	Male	White, nonH
4	GAD, PTSD, UDD, ADHD, ASD	15	Male	White, nonH
5	PTSD, MiID, ADHD	9	Male	Asian/PI, nonH
6	PTSD, ADHD, MiID	13	Male	White, nonH
7	DMDD, PTSD, ADHD, MiID	10	Male	White, nonH
8	DBD, ADHD, ASD, OSTSRD	8	Male	White, nonH
9	PTSD, MDD, ADHD, ASD	9	Male	White, nonH
10	GAD, PTSD, ADHD	7	Male	White, nonH
11	DBD, PTSD, DMDD, ADHD, ASD	12	Male	White, nonH
12	DBD, PTSD, MiID	13	Female	White, nonH
13	OSTSRD, DBD, MiID	14	Male	Black, nonH
14	PTSD, ADHD, ASD	11	Female	White, nonH
15	PTSD, ADHD, ASD	12	Transgender	White, nonH
16	DMDD, PTSD, ADHD, ASD	12	Transgender	White, nonH
17	PTSD, OCD, RAD, MiID, cluster B traits	15	Female	White, nonH
18	PTSD, ADHD, DBD	9	Male	Multiracial, H
19	PTSD, ADHD, ASD	12	Female	White, nonH
20	PTSD, ADHD, ASD, MiID	9	Female	White, nonH
21	MDD, OSTSRD, DMD, ASD	13	Female	White, nonH

Note: ID = participant identifier; DMDD = disruptive mood dysregulation disorder; PTSD = post-traumatic stress disorder; ADHD = attention deficit hyperactivity disorder; MiID = mild intellectual disability; ASD = autism spectrum disorder; DBD = disruptive behavior disorder; GAD = generalized anxiety disorder; UDD = unspecified depressive disorder; OSTSRD = other specified trauma- and stressor-related disorder; MDD = major depressive disorder; RAD = reactive attachment disorder; OCD = obsessive–compulsive disorder; Asian/PI = Asian/Pacific islander; nonH = non-Hispanic; H = Hispanic.

**Table 2:** Behavioral topographies and response to treatment.

Part ID	Topographies	FA Results	Treatment Evaluation	PB % Reduction	ACEs, Part 1	ACEs, Part 2
1	AGG, PD, NS, EL	AT	FCT AT	97.1	6	1
2	AGG, PD, NS, EL	AT	FCT ES	100	7	1
3	PD, NS	AT	FCT AT	100	6	2
4	NS	AT	FCT ES	0	4	1
5	PD, NS, EL	AT	FCT AT	100	1	1
			FCT ES	100		
6	NS	ES, TA	FCT ES	100	7	3
7	NS, IV	ES	FCT AT	0	2	2
	PD, NS, EL		FCT ES	0		
8	AGG, PD, NS, SIB	ES, TA	FCT ES	60	7	1
			FCT TA	100		
9	NS; EL	ES	FCT ES	52.9	5	1
			Ther Work	35.3		
10	NS	ES	FCT ES	100	5	2
11	AGG, PD, NS, SIB	ES, TA	FCT ES	100	4	1
12	AGG, PD, NS, SIB	Undiff	FCT ES	100	3	1
13	AGG, PD, NS, EL	ES, TA	FCT ES	100	5	6
14	NS	ES	FCT ES	68	4	2
			Ther Work	100		
15	AGG, PD, NS, EL	Undiff	FCT ES	0	7	5
16	NS (somatic complaints)	ES	Ther Work	40.3	7	6
17	NS; AGG; EL	TA	FCT ES	100	4	3
			FCT AT	100		
18	EL	Undiff	FCT ES	100	5	0
19	AGG, PD, NS, SIB	Undiff	FCT TA	100	5	3
			FCT ES	100		
20	PD, NS, EL	Undiff	FCT ES	0	7	2
21	PD, NS, EL	Undiff	FCT AT + TA	0	5	3
Average				72.4	5.0	2.2

Note: Pt ID = participant identifier; FA = functional analysis; PB % reduction = percentage of problem behavior reduction; ACEs = adverse childhood experiences; AGG = aggression; PD = property destruction; NS = negative statements; EL = elopement; IV = inappropriate vocalizations; SIB = self-injurious behaviors; AT = attention; ES = escape; TA = tangible; undiff = undifferentiated; FCT = functional communication training; ther = therapeutic work demands.

## Data Availability

Data are available upon request from the first author.

## Note

1 This manuscript alternates between person-first (e.g., “individuals with autism”) language and identity-first (e.g., “autistic individuals”) language to reflect the diversity of preferences within the disability community and the scholarly literature. This choice was made to respect both perspectives and acknowledge the evolving nature of inclusive language.
